# The LIM domain protein nTRIP6 modulates the dynamics of myogenic differentiation

**DOI:** 10.1038/s41598-021-92331-8

**Published:** 2021-06-18

**Authors:** Tannaz Norizadeh Abbariki, Zita Gonda, Denise Kemler, Pavel Urbanek, Tabea Wagner, Margarethe Litfin, Zhao-Qi Wang, Peter Herrlich, Olivier Kassel

**Affiliations:** 1 Institute for Biological and Chemical Systems-Biological Information Processing (IBCS-BIP), Karlsruhe Institute of Technology (KIT), Karlsruhe, Germany; 2 Leibniz Institute for Age Research (Fritz Lipmann Institute, FLI), Jena, Germany

**Keywords:** Differentiation, Cell biology

## Abstract

The process of myogenesis which operates during skeletal muscle regeneration involves the activation of muscle stem cells, the so-called satellite cells. These then give rise to proliferating progenitors, the myoblasts which subsequently exit the cell cycle and differentiate into committed precursors, the myocytes. Ultimately, the fusion of myocytes leads to myofiber formation. Here we reveal a role for the transcriptional co-regulator nTRIP6, the nuclear isoform of the LIM-domain protein TRIP6, in the temporal control of myogenesis. In an in vitro model of myogenesis, the expression of nTRIP6 is transiently up-regulated at the transition between proliferation and differentiation, whereas that of the cytosolic isoform TRIP6 is not altered. Selectively blocking nTRIP6 function results in accelerated early differentiation followed by deregulated late differentiation and fusion. Thus, the transient increase in nTRIP6 expression appears to prevent premature differentiation. Accordingly, knocking out the *Trip6* gene in satellite cells leads to deregulated skeletal muscle regeneration dynamics in the mouse. Thus, dynamic changes in nTRIP6 expression contributes to the temporal control of myogenesis.

## Introduction

Skeletal muscle regeneration relies on resident adult stem cells, the so-called satellite cells^1^. Upon muscle damage, these cells are activated and give rise to proliferating progenitor cells, the myoblasts. These then differentiate into committed precursor, the myocytes, which finally fuse together to form new multinucleated myofibres or fuse with existing myofibres^2–4^. Each step along this process of adult or regenerative myogenesis is tightly regulated by a network of transcription factors (TFs), including the myogenic TFs MYOD and myogenin and the myocyte enhancer factor 2 (MEF2) A B and C^5–7^. The temporal control of the activity of these TFs is essential to ensure the timely expression of their target genes and the subsequent progression through differentiation. One level of control is the expression of the TFs. For example, MYOD and myogenin are up-regulated in the early phases of muscle regeneration^8,9^ and in turn both these TFs drive the expression of MEF2C^10^, which is required in the late phases of myogenesis and in myofibre maturation^11,12^. However, the regulation of TF expression alone is not sufficient for a tight temporal control of differentiation. For example, despite its role in promoting terminal differentiation, MEF2C is already expressed in proliferating myoblasts^13,14^. Furthermore, a significant number of TFs involved in myogenesis do not show any regulation of their expression^15^. Therefore, not only the expression but also the transcriptional activity of TFs has to be regulated.

Our previous demonstration that nTRIP6, the nuclear isoform of the LIM domain protein TRIP6, acts as a co-repressor for MEF2C^16^ raises the hypothesis of its role in the regulation of myogenesis. TRIP6 belongs to the ZYXIN family of cytosolic LIM domain-containing proteins that regulate adhesion and migration^17,18^. Surprisingly, they also exert transcriptional co-regulator functions for various TFs, and have thus been proposed to shuttle from the cytoplasm to the nucleus^18–20^. Amongst these proteins a particular case is that of TRIP6^21–23^. We have reported that TRIP6 is not shuttling, but that its co-regulator functions are mediated by a shorter, exclusively nuclear isoform, which we termed nTRIP6^24–26^.

Here, we report that the expression of nTRIP6 is transiently increased at the transition between myoblast proliferation and differentiation and that nTRIP6 prevents premature differentiation. Thus, our data document the role of nTRIP6 in temporal control of myogenesis.

## Results

**Figure 1:**
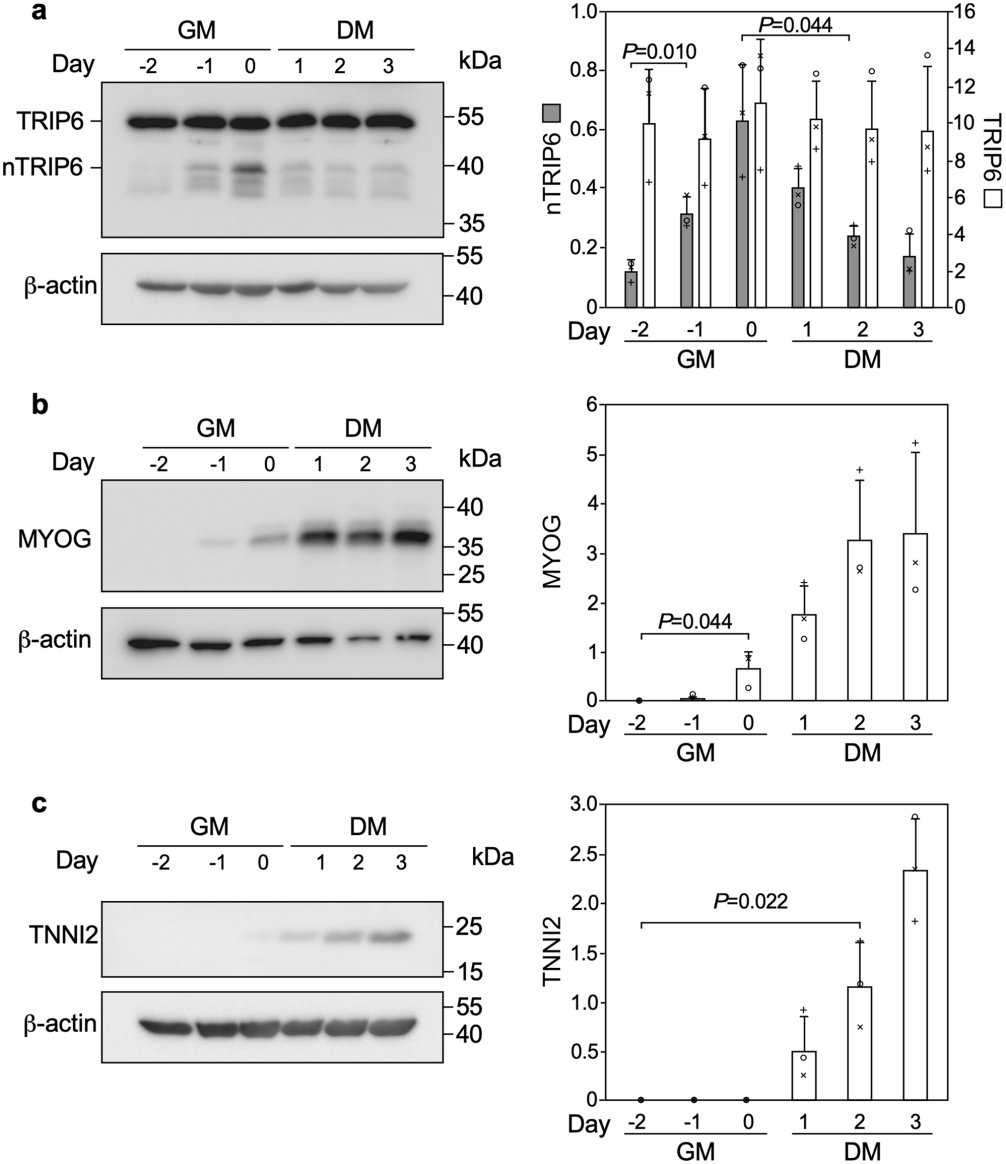
nTRIP6 expression is transiently increased during myoblast differentiation. C2C12 myoblasts lysates harvested at the indicated day of a differentiation experiment—day 0 corresponds to the switch from growth medium (GM) to differentiation medium (DM)—were subjected to Western blotting using the indicated antibodies. (**a**) Representative blots are shown. Full-length blots are presented in Supplementary Fig. S1. (**b**) The expression of TRIP6 and nTRIP6, as well as of MYOG and TNNI2 relative to the β-actin loading control are presented as mean ± SD of three independent experiments. Individual values are depicted by symbols, each representing an independent experiment.

**Figure 2:**
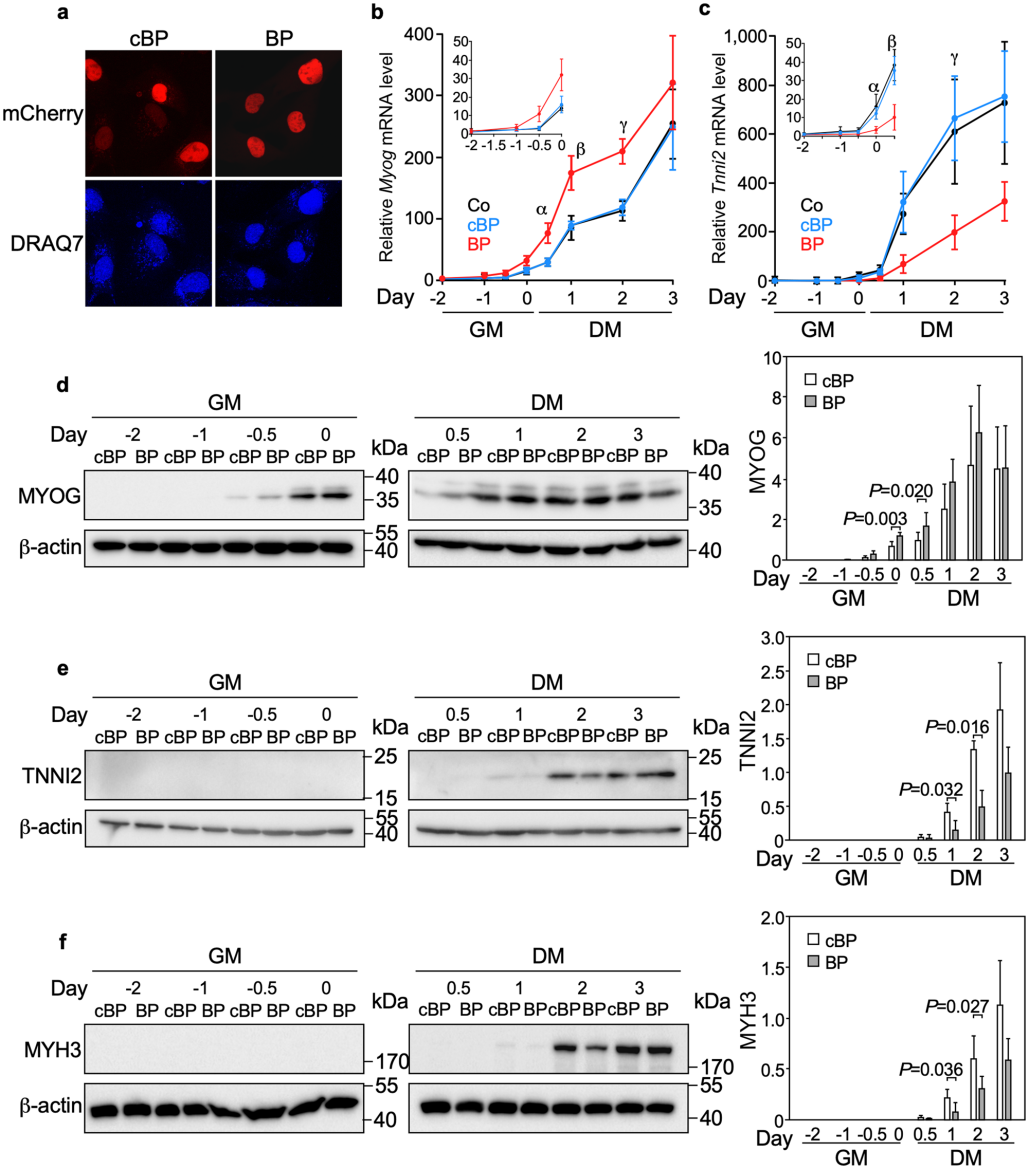
Blocking nTRIP6 function accelerates early and delays late differentiation. Control untransfected C2C12 cells (Co) or cells transfected with either the mCherry-tagged, nuclear targeted nTRIP6 blocking peptide (BP) or the control, scrambled version of the peptide (cBP) were subjected to a differentiation experiment. (**a**) Twenty-four hours after transfection cell nuclei were counterstained with DRAQ7 and imaged by confocal microscopy. Representative images are shown. (**b**,**c**) Cells were harvested at the indicated day and the relative levels of the *Myog* (**b**) and *Tnni2* (**c**) mRNAs were determined by reverse transcription and real-time PCR. Results are plotted relative to the expression of the *Rplp0* gene (mean ± SD of three independent experiments). Bonferroni corrected *P* values are for (**b**) α = 0.044, β = 0.027 and γ = 0.012 and for (**c**) α = 0.050, β = 0.053 and γ = 0.047. (**d**–**f**) C2C12 cells transfected with the BP or the cBP were harvested at the indicated day of a differentiation experiment. Cell lysates were subjected to Western blotting using the indicated antibodies. Representative blots are shown. Full-length blots are presented in Supplementary Fig. S5. The expression of MYOG, TNNI2 and MYH3 relative to the β-actin loading control are presented as mean ± SD of three independent experiments.

**Figure 3:**
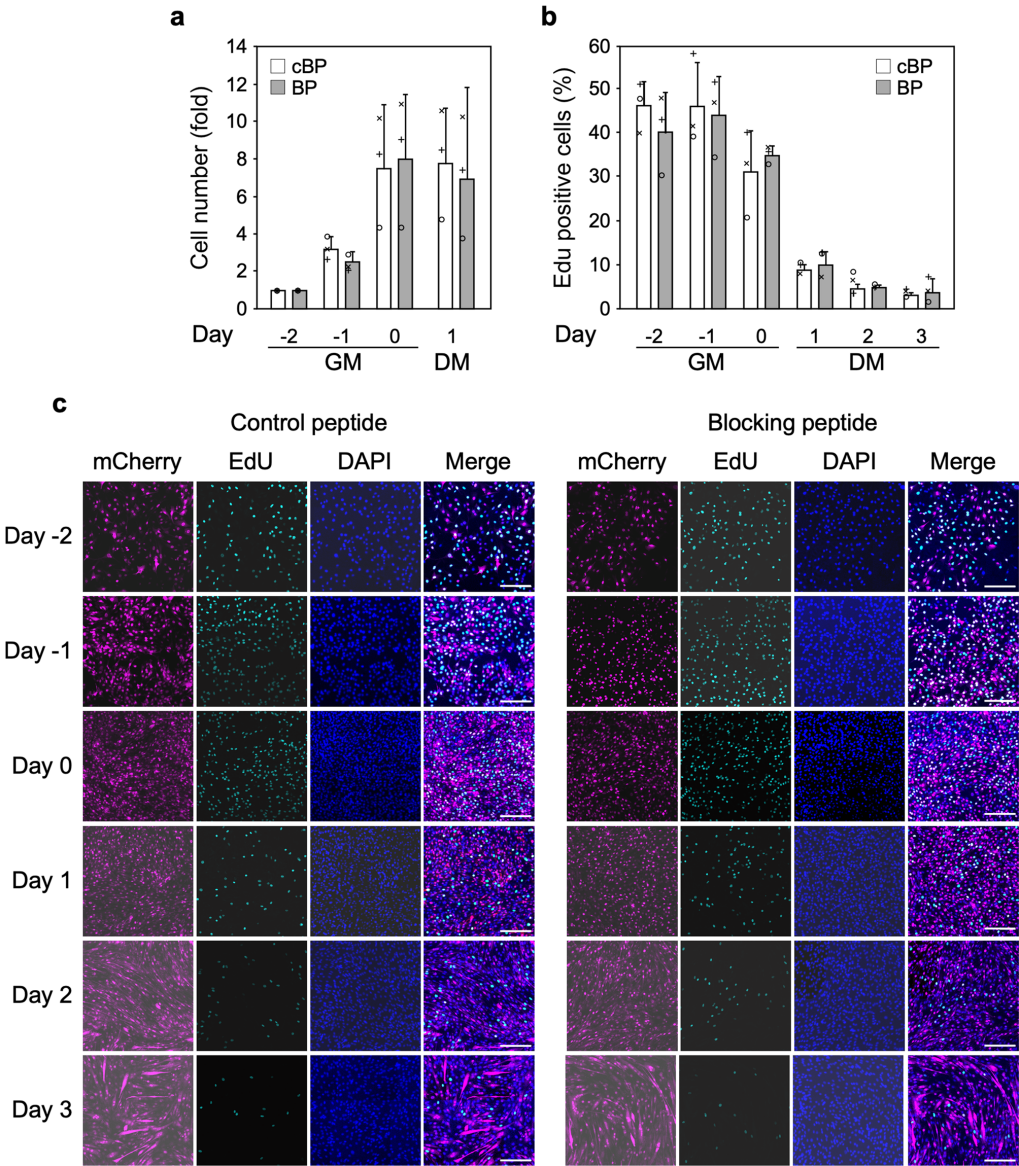
Blocking nTRIP6 function does not affect myoblast proliferation and cell cycle exit. C2C12 cells transfected with either the control (cBP) or the blocking peptide (BP) were subjected to a differentiation experiment. (**a**) Cells were counted at the indicated day and results are shown relative to the cBP-transfected cells at day-2 and are mean ± SD of three independent experiments. Individual values are depicted by symbols, each representing an independent experiment. (**b**, **c**) Cells were pulsed with EdU for 1 h at the indicated time point, fixed, stained for EdU incorporation and peptide expression (mCherry) and analysed by confocal microscopy. The percentage of EdU-positive nuclei among transfected cells (mCherry positive) is presented as mean ± SD of three independent experiments (**b**). Individual values are depicted by symbols, each representing an independent experiment. Representative images are shown (**c**, scale bar: 200 µm).

**Figure 4:**
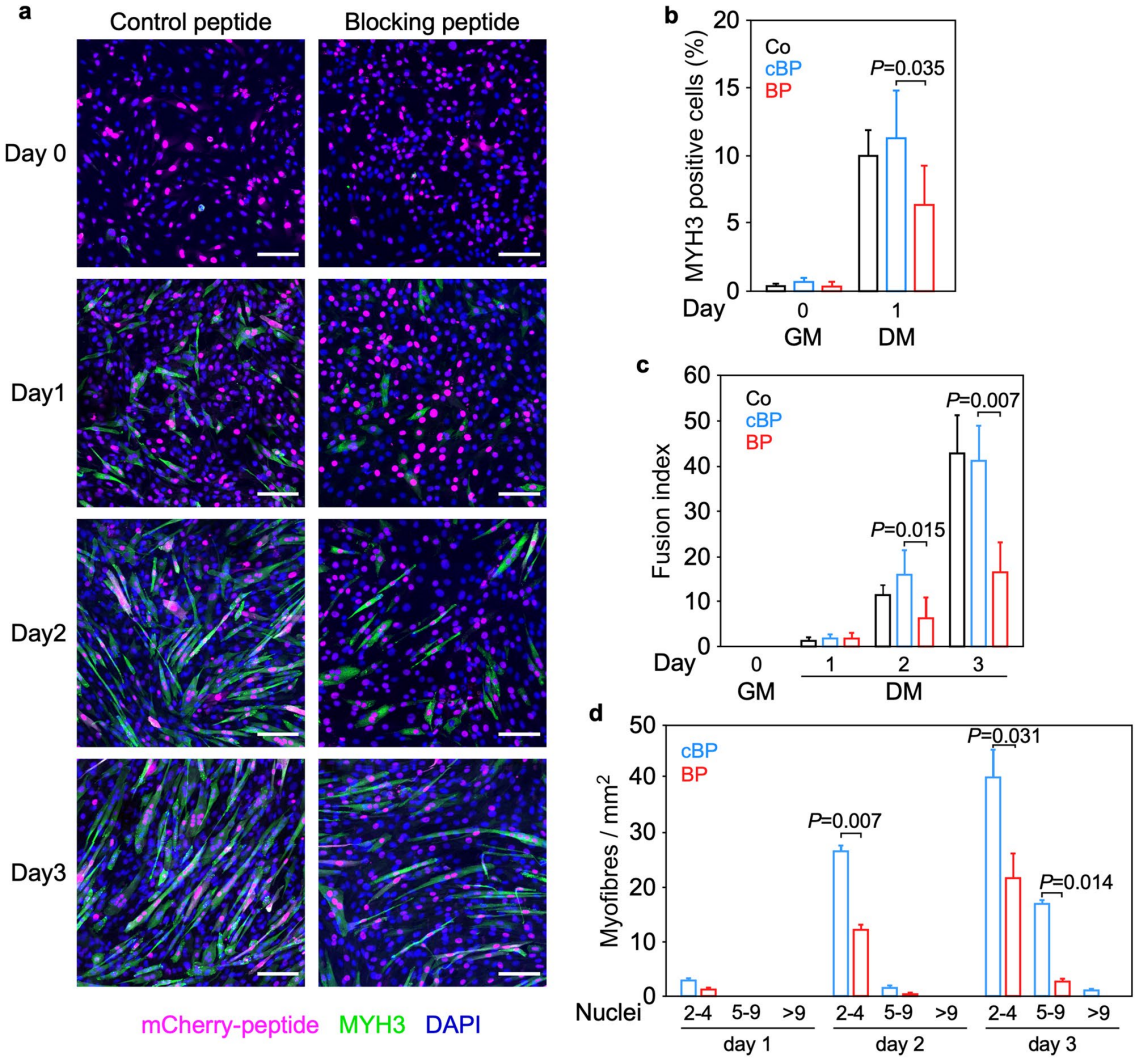
Blocking nTRIP6 function delays late differentiation and fusion. Control C2C12 cells transfected with either the mCherry-tagged, nuclear targeted nTRIP6 blocking peptide (BP) or the control, scrambled version of the peptide (cBP) were subjected to a differentiation experiment. Cells were fixed at the indicated day and subjected to immunofluorescence analysis using antibodies against MYH3 and mCherry. Nuclei were counterstained using DAPI. (**a**) Representative images are presented (scale bar 100 µm). (**b**) The percentage of MYH3 expressing cells among transfected cells (mCherry-positive), (**c**) the fusion index (percentage of mCherry positive nuclei within fused myotubes), and (d) the number of mCherry-positive nuclei per myofiber are presented as mean ± SD of three independent experiments.

As a first step in investigating a possible involvement of nTRIP6 in myogenic differentiation, we studied its expression during the in vitro differentiation of the C2C12 mouse myoblast cells line (Fig. 1). nTRIP6 expression was very low at the beginning of the proliferation phase. It then increased with cell density, to reach a maximum towards the end of the proliferation phase, when the cells were nearly fully confluent before the medium was changed to differentiation medium (day 0). This maximum coincided with the start of MYOG (also known as myogenin) expression, used as an indicator of early myocytic differentiation^27,28^. nTRIP6 levels were then decreased at time points when the expression of TNNI2, a late differentiation marker^29^ started to increase and when myocyte fusion typically occurred (see Fig. 4 for fusion). Strikingly, the expression of the large cytosolic isoform TRIP6 remained constant (Fig. 1) and *Trip6* mRNA levels were not increased (Supplementary Fig. S2) throughout the entire course of the experiment. The dynamics of its expression may suggest a regulatory role for nTRIP6 in the transition between proliferation and differentiation. We first investigated the putative role of nTRIP6 in differentiation. To address this question, we made use of a genetically encoded, nuclear-targeted, mCherry tagged blocking peptide that selectively inhibits the function of nTRIP6 in the nucleus without interfering with TRIP6 in the cytosol, as well as a scrambled version of the peptide as a control^16,30^. Upon transfection in C2C12 cells, the peptides were exclusively located in the nucleus (Fig. 2a). Furthermore, the blocking peptide did not affect the cytosolic function of TRIP6, i.e. adhesion^31,32^, whereas an siRNA which knocked-down both isoforms did (Supplementary Fig. S3), confirming the selectivity of the blocking peptide. In the C2C12 cells expressing the blocking peptide, *Myog* mRNA was expressed earlier in the proliferation phase and remained more elevated during the differentiation phase than in control cells expressing the scrambled version of the peptide (Fig. 2b). Conversely, the expression of *Tnni2* was delayed in cells expressing the blocking peptide as compared to the control cells (Fig. 2c). These effects were also observed at the protein level: the blocking peptide increased the expression of MYOG at early time points (Fig. 2d) and, at later time points decreased that of TNNI2 (Fig. 2e), as well as that of MYH3 (embryonic myosin heavy chain) used as another marker of late differentiation (Fig. 2f). Given the known co-regulation of cell cycle exit and entry into differentiation^33^, we also assessed proliferation. There was no difference in the proliferation (Fig. 3a) and in the kinetics of cell cycle exit assessed by EdU pulse labelling (Fig. 3b,c) between myoblasts transfected with the nTRIP6 blocking peptide and myoblasts transfected with the control peptide. We then studied the effect of the peptide on late differentiation and fusion. As another index of late differentiation, we quantified the number of cells expressing MYH3 upon transfection of the peptides (Fig. 4a,b). At day 0, just before the induction of differentiation, very few cells expressed MYH3, as expected. Twenty-four hours later (day 1), about 10% of the cells transfected with the control peptide expressed MYH3. This number was significantly decreased upon transfection of the nTRIP6 blocking peptide (Fig. 4b). Finally, cell fusion, which started at day 2 and strongly increased at day 3 in cells transfected with the control peptide, was significantly inhibited in cells transfected with the blocking peptide (Fig. 4c,d). Thus, blocking nTRIP6 function in myoblasts accelerates early but delays late myocytic differentiation and impairs myocyte fusion.

**Figure 5:**
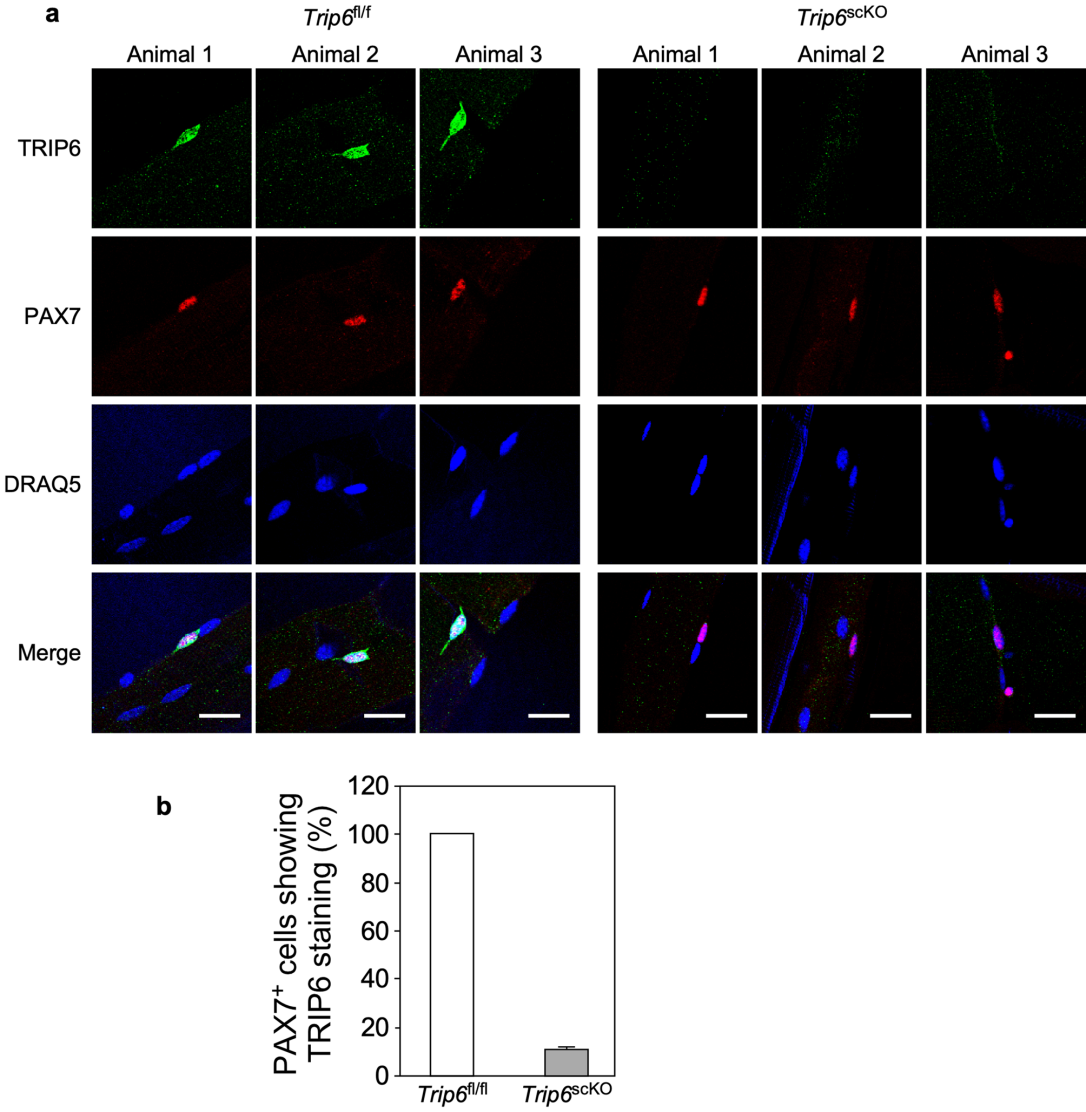
Recombination efficiency in satellite cells and loss of TRIP6/nTRIP6 expression in *Trip6*^scko^ myogenic cells. Myofibres with the associated satellite cells isolated from the Extensor digitorum longus of *Trip6*^fl/fl^ and *Trip6*^scko^ mice were subjected to immunofluorescence analysis using anti-TRIP6/nTRIP6 and anti-PAX7 antibodies. Nuclei were counterstained using DRAQ5. (**a**) Representative images are shown (scale bar: 20 µm). (**b**) The percentage of satellite cell (PAX7-positive) showing TRIP6 / nTRIP6 immunoreactivity is presented as mean ± SD (3 animals per genotype, 10 myofibres per animal).

**Figure 6:**
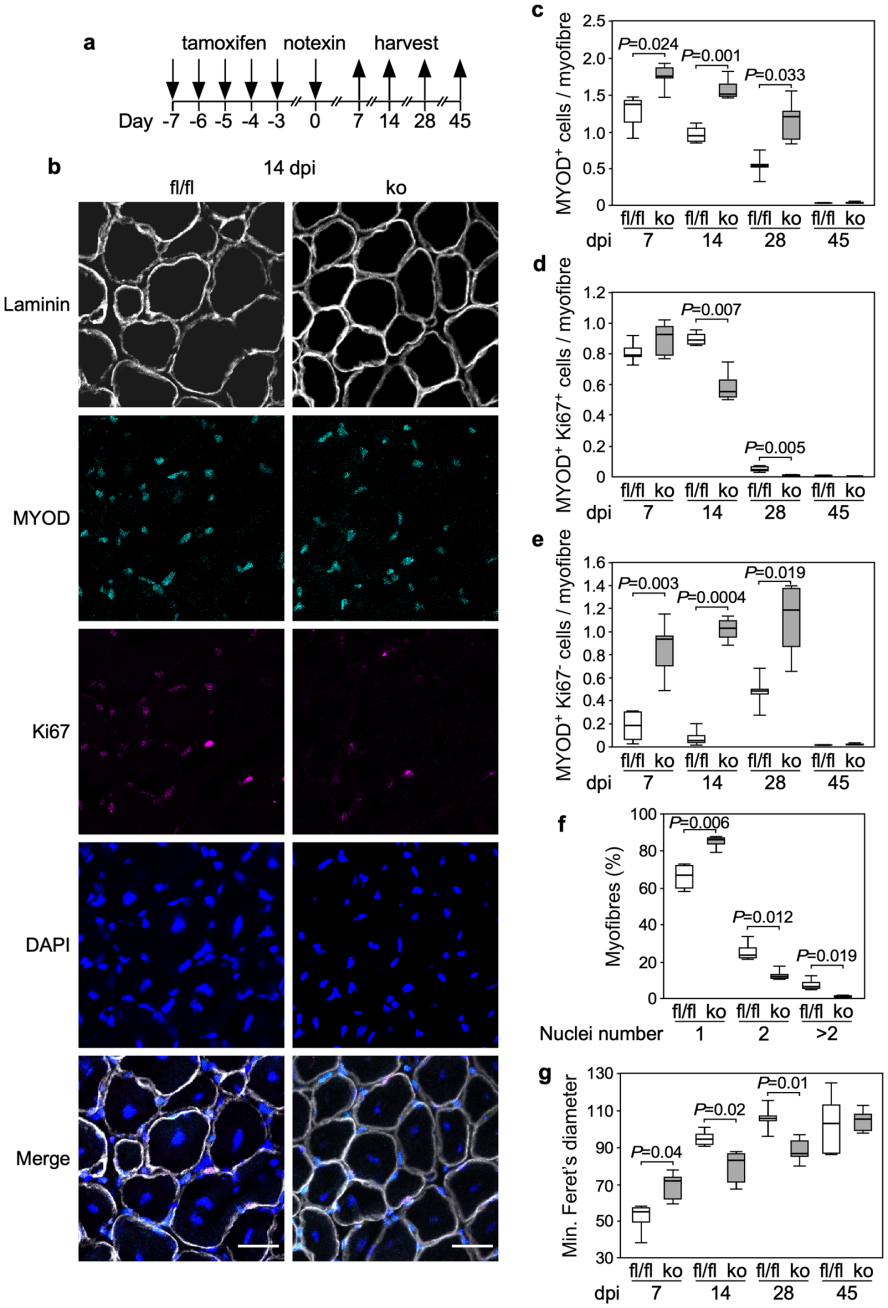
Skeletal muscle regeneration in *Trip6* knockout mice. (**a**) Schematic representation of the protocol. The *Trip6* gene was knocked out in satellite cells by intraperitoneal injection of tamoxifen in *Trip6*^fl/fl^; *Pax7*^Cre-ERT2/wt^ mice and muscle degeneration was induced by injection of notexin in the *M. soleus*. (**b-g**) The regenerating and uninjured contralateral muscles of *Trip6*^scko^ (ko) and *Trip6*^fl/fl^ control animals (fl/fl) were harvested at the indicated day post-injury (dpi). Muscle sections were stained with anti-Laminin, anti-MYOD and anti-Ki67 antibodies and counterstained with DAPI. (**b**) Representative images at 14 dpi are shown (the full set is presented in Supplementary Fig. S6; scale bar: 30 µm). (**c**) Number of MYOD-positive mononuclear cells normalized to the number of myofibres; (**d**) Number of MYOD-positive mononuclear cells expressing Ki67 normalized to the number of myofibres; (**e**) Number of MYOD-positive mononuclear cells not expressing Ki67 normalized to the number of myofibres; (**f**) distribution of the number of centrally located nuclei detected on myofibres cross-sections; (**g**) Minimum Feret’s diameter of the regenerating myofibres expressed as percent of the contralateral uninjured muscle. Bonferroni corrected *P* values are presented (5 animals per group).

**Figure 7:**
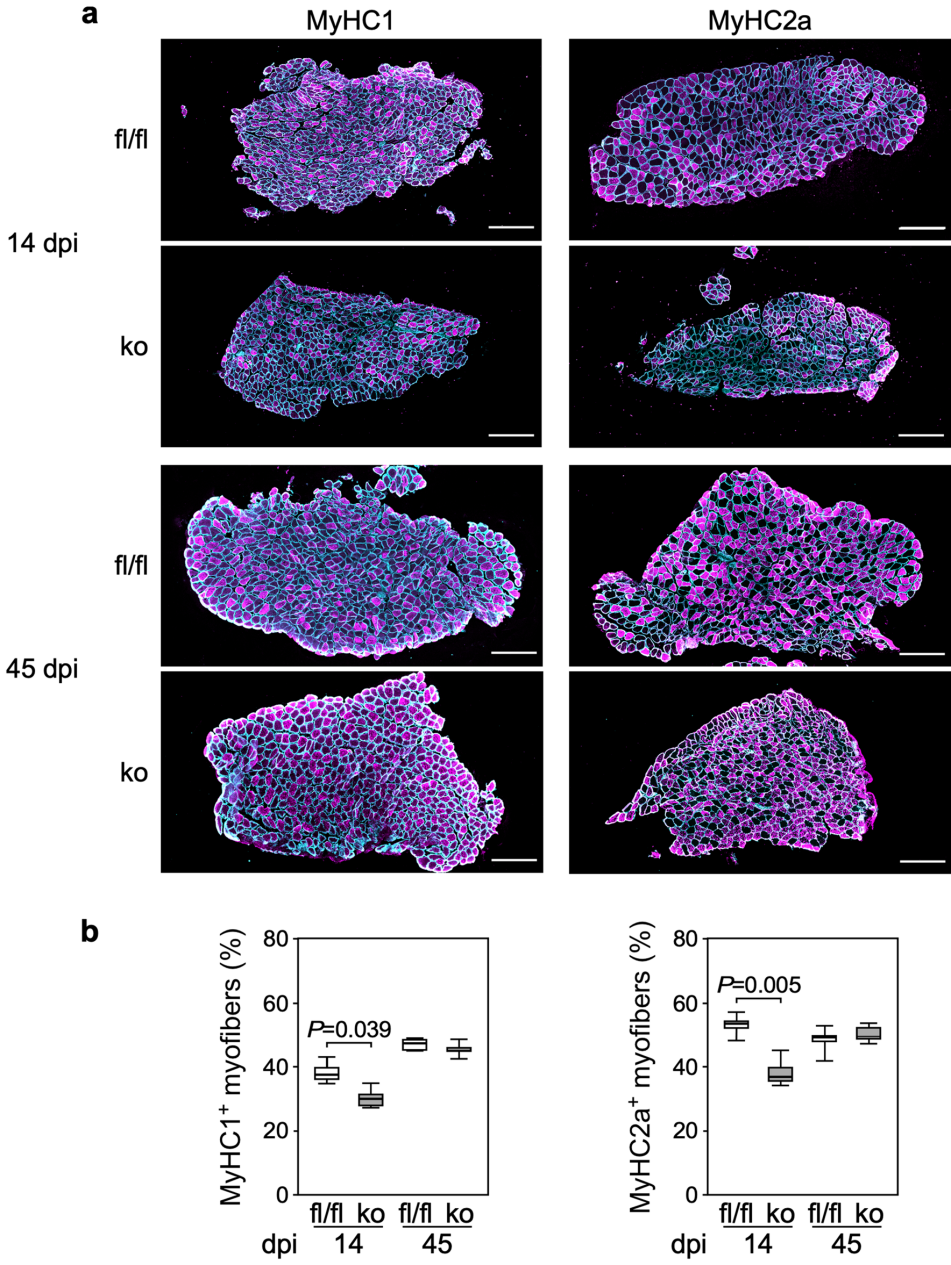
Expression of myosin heavy chains. Sections of regenerating *Trip6*^scko^ (ko) and *Trip6*^fl/fl^ control (fl/fl) muscles harvested at the indicated day post- injury (dpi) were stained with an anti-Laminin antibody (cyan) together with either an anti-MyHC1 or an anti-MyHC2a antibodiy (magenta) as indicated. (**a**) Representative images are shown (scale bar: 250 µm). (**b**) The number of myofibres positive for MyHC1 and MyHC2a is presented as percent of the total number of myofibres. Bonferroni corrected *P* values are presented (5 animals per group).

To investigate the relevance of these findings for muscle regeneration in vivo, we generated a mouse line, C57BL/6J-*Trip6*^Tm(loxP)^ hereafter referred to as *Trip6*^fl/fl^, in which the *Trip6* gene is conditionally targeted by flanking exons 2 to 9 by loxP sites^34^. These mice were crossed with C57BL/6J-*Pax7*^tm1(Cre-ERT2)Gaka^ mice (short name *Pax7*^Cre-ERT2^), in which the tamoxifen-inducible version of Cre-recombinase (Cre-ERT2) was knocked into the *Pax7* locus^35^. Treatment of the *Trip6*^fl/fl^;*Pax7*^Cre-ERT2/wt^ offspring with tamoxifen results in the deletion of *Trip6* only in satellite cells. These animals are hereafter termed *Trip6*^scko^. We verified the efficiency of tamoxifen-induced recombination by monitoring TRIP6 immunoreactivity in satellite cells associated with isolated myofibres. Whereas 100% of PAX7+ satellite cells from *Trip6*^*fl/fl*^ mice showed TRIP6 immunoreactivity, it was decreased to about 10% of the satellite cells from *Trip6*^*scko*^ animals treated with tamoxifen for 5 consecutive days (Fig. 5). Then, the animals were subjected to a muscle injury by direct injection of notexin into the soleus muscle^36^ and regeneration was followed over time (Fig. 6; Supplementary Fig. S6). As an index of myogenic differentiation during regeneration, we counted the number of mononuclear cells expressing MYOD (Fig. 6c), which includes both proliferating myoblasts and differentiated myocytes^8,9^. Seven days post injury (dpi) the number of MYOD-positive cells was significantly higher in the regenerating muscle of *Trip6*^*scko*^ animals than in the muscle of the *Trip6*^*fl/fl*^ control mice. This difference was also observed at 14 dpi and 28 dpi. At 45 dpi, the number of MYOD-positive cells had strongly decreased and was not different between the two genotypes (Fig. 6c). This increase in the number of MYOD^+^ cells could reflect an increased myoblast proliferation or an increase in the number of unfused differentiated myocytes. Therefore, we also quantified the number of MYOD^+^ cells expressing and not expressing the proliferation marker Ki67 (Fig. 6d,e). The number of proliferating myoblasts (MYOD^+^/Ki67^+^) was not different between the *Trip6*^*scko*^ and *Trip6*^*fl/fl*^ regenerating muscles at 7 dpi. This number was significantly lower in the *Trip6*^*scko*^ than in the *Trip6*^*fl/fl*^ muscles at both 14 and 28 dpi (Fig. 6d). Conversely, at 7, 14 and 28 dpi, the number of post-mitotic myocytes (MYOD^+^/Ki67^−^) was significantly higher in the *Trip6*^*scko*^ than in the *Trip6*^*fl/fl*^ muscles (Fig. 6e). Thus, the higher number of MYOD-expressing cells in the *Trip6*^*scko*^ muscle is not caused by an increased proliferation but to an accumulation of unfused myocytes. Indeed, we observed an inhibition of myocyte fusion, as indicated by a lower number of regenerating myofibres with multiple centrally located nuclei detected on the cross-sections of *Trip6*^*scko*^ muscles as compared to *Trip6*^*fl/fl*^ regenerating muscles (Fig. 6f). Given that an inhibition of myocyte fusion might lead to reduced myofibre size, we assessed the size of the regenerated myofibres by measuring their minimum Feret’s diameter (Fig. 6g). At 7 dpi, the minimum Feret’s diameter of the myofibres was larger in *Trip6*^*scko*^ than in *Trip6*^*fl/fl*^ muscles. However, this was the opposite at 14 and 28 dpi, with larger myofibres in *Trip6*^*fl/fl*^ muscles. Finally, at 45 dpi, there was no more difference in the minimum Feret’s diameter of the regenerated myofibres in both genotypes (Fig. 5g). Finally, we counted the number of regenerated myofibres expressing the slow-type myosin heavy chains MyHC1 and MyHC2a (Fig. 7) as an indication of their maturation. At 14 dpi, the number of both MyHC1^+^ and MyHC2a^+^ myofibres was lower in *Trip6*^*scko*^ than in *Trip6*^*fl/fl*^ muscles. This difference was not present anymore at 45 dpi. Thus, very similar to our observations in vitro, the loss of nTRIP6 expression in satellite cells results in a deregulation of the dynamics of muscle regeneration.

## Discussion

We have here unravelled the role of nTRIP6 in the temporal control of myogenesis. nTRIP6 expression is transiently up-regulated at the transition between myoblast proliferation and myocytic differentiation. In turn, nTRIP6 temporarily represses myoblast differentiation, allowing proper myocyte differentiation and fusion at later stages.

Our observation that nTRIP6 expression increases at the transition between myoblast proliferation and early differentiation suggested a function at this critical time point. Indeed, blocking nTRIP6 function in the nucleus using a nuclear-targeted peptide^16,30^ accelerated early myocytic differentiation but did not have any effect on proliferation and cell cycle exit. Thus, the function of nTRIP6 at this stage of myogenesis is to prevent premature differentiation. We have recently reported that nTRIP6 acts as a transcriptional co-repressor for MEF2C^16^, a transcription factor involved in late differentiation and fusion^11,12^, which is however already expressed during the myoblast proliferation phase^13,14^. Notably*, Myog* is a known MEF2C target gene^37^ and our result of an accelerated/increased expression of *Myog* upon blocking nTRIP6 function is fully in line with the co-repressor function of nTRIP6 for MEF2C. Thus, the transient increase in nTRIP6 expression at early stages of myogenesis may serve to prevent premature differentiation at these stages at least in part by repressing the transcriptional activity of MEF2C. The lack of nTRIP6 activity delayed the expression of TNNI2 and MYH3, which are also MEF2C target genes^38,39^. One would have rather expected an increased expression of these genes upon blocking the function of nTRIP6. Thus, the effect of nTRIP6 on the expression of these late differentiation cannot be attributed to the co-repressor function for MEF2C. Our results also show that blocking nTRIP6 functions inhibits late differentiation and fusion. However, the expression of nTRIP6 is no more elevated when fusion starts at day 2. Therefore, the inhibitory effect of blocking nTRIP6 function on late differentiation and fusion is most likely an indirect consequence of the lack of nTRIP6 activity at earlier time points, leading to a deregulated early differentiation. The in vivo relevance of these findings is confirmed by our results on muscle regeneration in mice in which *Trip6* is knocked out in satellite cells. For example, we observed an increase in the number of MYOD-expressing cells in *Trip6*^scko^ regenerating muscle. Given that both myoblasts and myocytes express MYOD^8,9^, this increase could be due to either an increased myoblast proliferation or a decreased myocyte fusion. However, we did not observe an increased but rather a decreased number of proliferating myoblasts in the *Trip6*^scko^ regenerating muscle, associated with an increased number of myocytes. This observation matches the accelerated early myocytic differentiation that we observed upon blocking nTRIP6 function in vitro. Furthermore, the increased number of myocytes associated with a reduced number of centrally located nuclei and with a delay in the increase in myofibre size in the *Trip6*^scko^ regenerating muscle confirms a delay in myocyte fusion. Therefore, the deregulated dynamics of muscle regeneration in the *Trip6*^scko^ animal is very reminiscent of the deregulated dynamics of in vitro myogenesis that we observed upon blocking nTRIP6 function. In the *Trip6*^scko^ knock out animals the expression of both the long cytosolic isoforms (TRIP6) and short nuclear isoform (nTRIP6) is lost. Thus, the observed regeneration phenotype could be due to the loss of either nTRIP6 in the nucleus or TRIP6 in the cytosol or both. The main function of the cytosolic isoform TRIP6 is to regulate adhesion and migration^21,23^, a property shared by other members of the ZYXIN family of focal adhesion LIM domain proteins^40^. However, no general phenotype was observed upon the total deletion of the *Trip6* gene in the mouse, as could have been expected from the loss of such an important function^34^. This observation, together with the absence of overt phenotypes in mice with deletion of the genes for ZYXIN^41^, LPP^42^, Ajuba^43^, LIMD1^44^, and Migfilin^45^, strongly suggests a redundancy in the function of TRIP6 and other family members in the cytosol. It is therefore unlikely that the muscle regeneration phenotype that we observed in *Trip6*^scko^ is due to the loss of the cytosolic function of TRIP6. Furthermore, the striking similarities between the in vivo phenotype and the in vitro effects of the nTRIP6 blocking peptide, which is targeted to the nucleus and can thus only block the function of nTRIP6, very strongly suggests that the delayed muscle regeneration in the knock out is a consequence of a loss of nTRIP6. Therefore, nTRIP6 modulates the dynamics of myogenesis and of muscle regeneration by preventing premature differentiation.

## Methods

### Cell lines, transfection and cellular assays

C2C12 myoblasts (obtained from ATCC, LGC Standards GmbH, Wesel, Germany) were cultured in Dulbecco’s modified Eagle’s medium (DMEM) supplemented with 10% foetal calf serum (FCS) and were routinely checked for mycoplasma contamination. We used a standardized protocol for the differentiation of C2C12 myoblasts. Cells were seeded at a density of 5 × 10^3^ cells/cm^2^ in growth medium (GM, 10% FCS-containing DMEM) at day-3, relative to the induction of differentiation at day 0. When cells reached confluence at day 0, differentiation was induced by changing the medium to differentiation medium (DM, 2% horse serum-containing DMEM), which was then replaced every day. For transfection, the pcDNA3.1 expression vectors for the mCherry-tagged, nuclear targeted nTRIP6 blocking peptide and the scrambled control peptide have been described^30^. Transfection was performed by nucleofection using the Nucleofector Kit V (Lonza, Cologne, Germany), reaching a transfection efficiency of 80% (5% confidence interval: 74–86%, n = 7), and the cells were seeded for differentiation as described above.

The synthetic siRNA duplex targeting mouse *Trip6* mRNA^25^, and a control siRNA duplex targeting dsRed^46^ were purchased from Eurofins MWG Operon (Ebersberg, Germany). siRNAs were transfected at a concentration of 20 mM into C2C12 cells using *Trans*IT-X2 (Mirus Bio LLC, Madison, WI). The sequences of the siRNAs are provided in Supplementary Table S1.

For adhesion assays, plasmid or siRNA transfected C2C12 myoblasts were trypsinized and re-seeded 48 h post-transfection onto collagen-coated glass coverslips at a density of 2.5 × 10^4^ viable (trypan blue exclusion) cells/cm^2^ and allowed to adhere for 1 h. Non-adhered cells were washed away and adhered cells were fixed for 10 min in 10% formalin. Nuclei were stained with DRAQ7 (Biostatus Ltd., Shepshed, UK), imaged and counted (see below).

### RNA isolation and quantitative real-time PCR (qRT-PCR)

Total RNA was extracted using PeqGOLD TriFast (Peqlab Biotechnologie, Erlangen, Germany) and reverse-transcribed into cDNA. *Myog* (myogenin) and *Tnni2* mRNAs, as well as the transcript of the large ribosomal subunit P0 gene (*Rplp0*) used for normalization, were quantified by real-time PCR using the ABI Prism Sequence Detection System 7000 (Applied Biosystems, Foster City, CA). The primers (Invitrogen) are described in Supplementary Table S2.

### Western blotting

Western blot analyses were performed using a custom-made rabbit anti-TRIP6 monoclonal antibody^16^, a rabbit anti-mCherry antibody (ab167453, abcam, Cambridge, UK), a rabbit anti-MYOG antibody (ab124800, abcam), a Goat anti-TNNI2 antibody (EB12036, Everest Biotech, Upper Heyford, UK), a mouse anti-beta-actin (AC-15, Sigma-Aldrich) and a rabbit anti-glucocorticoid receptor (GR) antibody (sc1002, Santa Cruz, Heidelberg, Germany) which was used as a loading control. Signals were detected by enhanced chemoluminescence using the ChemiDoc Touch Imaging System (BioRad laboratories, Munich, Germany). Signal quantification was performed within the linear range of detection using the Image Lab software (Bio-Rad laboratories). Linear brightness and contrast adjustments were made for illustration purposes only after the analysis had been made.

### Animals

Mice were housed and maintained under specific pathogen-free conditions in facilities approved by the Regierungspräsidium Karlsruhe. All animals were handled according to German regulations for animal experimentation and experiments were authorized by the Regierungspräsidium Karlsruhe (authorizations 35-9185.81/G-181/09 and 35-9185.81/G-261/15). C57BL/6 J-*Pax7*^tm1(Cre-ERT2)Gaka^ mice, hereafter referred to as *Pax7*^Cre-ERT2^, in which the tamoxifen-inducible version of Cre-recombinase (Cre-ERT2) was knocked in the *Pax7* locus^35^ were kindly provided by Gabrielle Kardon (University of Utah, Salt Lake City, UT) and Daniel Metzger (IGBMC, Strasbourg, France). The generation of the *Trip6* floxed mouse line (C57BL/6 J-*Trip6*^Tm(loxP)^ hereafter referred to as *Trip6*^fl/fl^) has been described^34^. *Trip6*^fl/fl^ were crossed to *Pax7*^Cre-ERT2^ and *Trip6*^fl/fl^ and *Trip6*^fl/fl^; *Pax7*^Cre-ERT2/wt^ offspring were identified by PCR genotyping on tail DNA. The genotyping primers (Invitrogen) are described in Supplementary Table S3. Experiments were performed using 6- to 8-week-old male animals. To selectively knock out *Trip6* in satellite cells, mice were injected intraperitoneally on 5 consecutive days with 5 μl/g body weight Tamoxifen (Sigma-Aldrich) from a 10 mg/ml stock solution in peanut oil. Three days after the last injection, soleus muscle degeneration was induced by notexin injection as described^36^. Briefly, animals were anesthetized by an intraperitoneal injection of Ketamin (100 mg/kg body weight) and Xylasin (16 mg/kg body weight). Notexin (10 μl of a 5 ng/μl solution in PBS) was injected unilaterally in the soleus muscle exposed through a small cutaneous incision. Animals were sacrificed by cervical dislocation 7, 14, 28 and 45 days post-injury and both the regenerating and contralateral soleus muscles were dissected. Group size (5 animals) was determined by power analysis based on effect sizes reported in the literature. No randomization was performed given that our aim was to compare mice with a deletion of *Trip6* in satellite cells (*Trip6*^scko^) with control animals (*Trip6*^fl/fl^). Although these experiments were not blinded sensu stricto, for the analyses the samples were labelled only with a code number.

For myofibre isolation, Extensor digitorum longus muscles were dissected and digested for 1 h at 37 °C in DMEM containing 0.2% collagenase II (Sigma-Aldrich). Myofibres were then dissociated by trituration through a Pasteur pipette and isolated manually under a stereomicroscope.

### Immunofluorescence, microscopy and image analysis

Immunofluorescence analysis was performed on C2C12 cells grown and differentiated on glass coverslips coated with collagen Type I (Sigma-Aldrich), fixed for 10 min in 10% formalin, permeabilized for 10 min in 0.5% Triton X-100 in PBS and blocked for 1 h in 5% BSA in PBS, as well as on 10 µm thick cryosections in the mid-belly of the soleus muscle fixed for 5 min in 2% paraformaldehyde, permeabilized for 10 min in 0.5% Triton X-100 in PBS and blocked for 1 h in 5% BSA in PBS. The same procedure was used for isolated myofibres. The primary antibodies were a rabbit anti-mCherry (ab167453, abcam, Cambridge, UK), a mouse anti-MYH3 (F1.652-b, Developmental Studies Hybridoma Bank, deposited by Blau H.M.), a rat anti-laminin (ab11576, abcam), a rabbit anti-MYOD (PA5-23078, ThermoFisher Scientific), an Alexa Fluor 647-conjugated rabbit anti-Ki67 (12075, Cell Signaling), a rabbit anti-TRIP6/nTRIP6^16^, a mouse anti-MYHC1 (BA-D5, Developmental Studies Hybridoma Bank, deposited by Schiaffino S.), a mouse anti-MYHC2a (SC-71, Developmental Studies Hybridoma Bank, deposited by Schiaffino S.) and a mouse anti-PAX7 (Developmental Studies Hybridoma Bank, deposited by Kawakami, A.). The secondary antibodies were an Alexa Fluor 488-conjugated anti-rabbit, an Alexa Fluor 546-conjugated anti-rat, an Alexa Fluor 546-conjugated anti-mouse and an Alexa Fluor 488-conjugated anti-mouse antibodies (Invitrogen). Nuclei were counter-stained with either DAPI (Sigma-Aldrich) or DRAQ5 (Biostatus Ltd., Shepshed, UK). EdU (5-ethynyl-2′-deoxyuridine) staining was performed using the EdU Click-488 kit (Roth, Karlsruhe, Germany). Briefly, C2C12 myoblasts transfected with either the nTRIP6 blocking peptide (BP) or the control peptide (cBP) and grown on coverslips were pulsed for 1 h with 10 µM EdU and then fixed for 15 min with 2% paraformaldehyde, permeabilized for 20 min with 0.5% Triton X-100 in PBS and blocked for 1 h in 5% BSA in PBS. Incorporated EdU was reacted with 6-FAM-Azide as recommended by the manufacturer. Cells were then stained with a rabbit anti-mCherry antibody (ab167453, abcam) and an Alexa Fluor 546-conjugated anti-rabbit secondary antibody. Nuclei were counterstained with DAPI. Cells and muscle sections were imaged by confocal microscopy on a Zeiss LSM 800 (Zeiss, Jena, Germany). Cells images were acquired in tiling mode using a 10×/0.3 Plan-Neofluar objective resulting in 3 × 2 mm^2^ images. Muscle sections were imaged using a 20×/0.8 Plan-Apochromat objective in order to image the entire section. Images were analysed using Fiji^47^. For the cell experiments, the number of transfected cell nuclei (mCherry positive) and the number of MYH3 or of EdU positive cells were determined by a combination of automated segmentation and manual counting in order to calculate the number of positive cells among transfected cells. The total number of counted cells was in the range of 700–3000 per sample. The fusion index was calculated as the percentage of mCherry positive nuclei within fused myotubes. For muscle sections, the regenerated myofibres (central nuclei) were segmented using the Laminin staining in order to determine their number and minimum Feret’s diameter as a measure of muscle fibre cross-sectional size^48^. The number of Ki67 positive nuclei and the total number of nuclei were determined by automated segmentation whereas the number of nuclei within myofibres was determined by manual counting, in order to calculate the percentage of non-myofibre nuclei expressing Ki67. The number of MYOD positive cells normalized to the number of myofibres was determined by manually counting the MYOD positive nuclei, excluding those within myofibres. Linear brightness and contrast adjustments were made for illustration purposes, but only after the analysis had been made.

### Statistical analysis

Statistical analyses were performed using R. Where indicated, significant differences were assessed by two-sided t-test analysis, with values of *P* < 0.05 sufficient to reject the null hypothesis. A Bonferroni correction was applied when multiple comparisons were performed.

## Supplementary Information

Supplementary Information.

## Data Availability

The datasets generated during and/or analysed during the current study are available from the corresponding author on reasonable request.
