# Supplementary material for: The LIM domain protein nTRIP6 modulates the dynamics of myogenic differentiation
Source: Sci Rep. 2021 Jun 18;11:12904. doi: 10.1038/s41598-021-92331-8 (PMC8213751; doi:10.1038/s41598-021-92331-8)
Supplement: Supplementary file 1 — Supplementary Information. [file 41598_2021_92331_MOESM1_ESM.pdf]

The LIM domain protein nTRIP6 modulates the dynamics of myogenic differentiation

Tannaz Norizadeh Abbariki[1,3], Zita Gonda[1,3], Denise Kemler[1], Pavel Urbanek[2], Tabea Wagner[1], Margarethe Litfin[1], Zhao-Qi Wang[2], Peter Herrlich[2], Olivier Kassel[1]*

[1] Karlsruhe Institute of Technology (KIT), Institute for Biological and Chemical Systems - Biological Information Processing (IBCS-BIP), Karlsruhe, Germany
[2] Leibniz Institute for Age Research (Fritz Lipmann Institute, FLI), Jena, Germany

[3] These authors contributed equally.

* Corresponding author:
e-mail: olivier.kassel@kit.edu

Fig. 1a

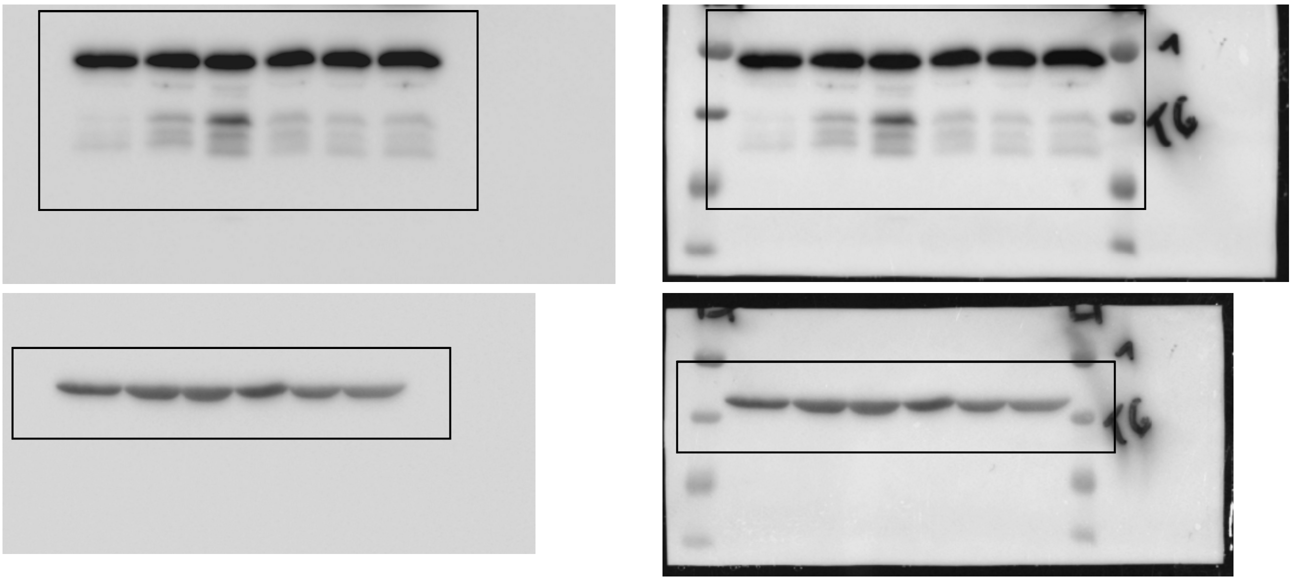

Fig. 1b

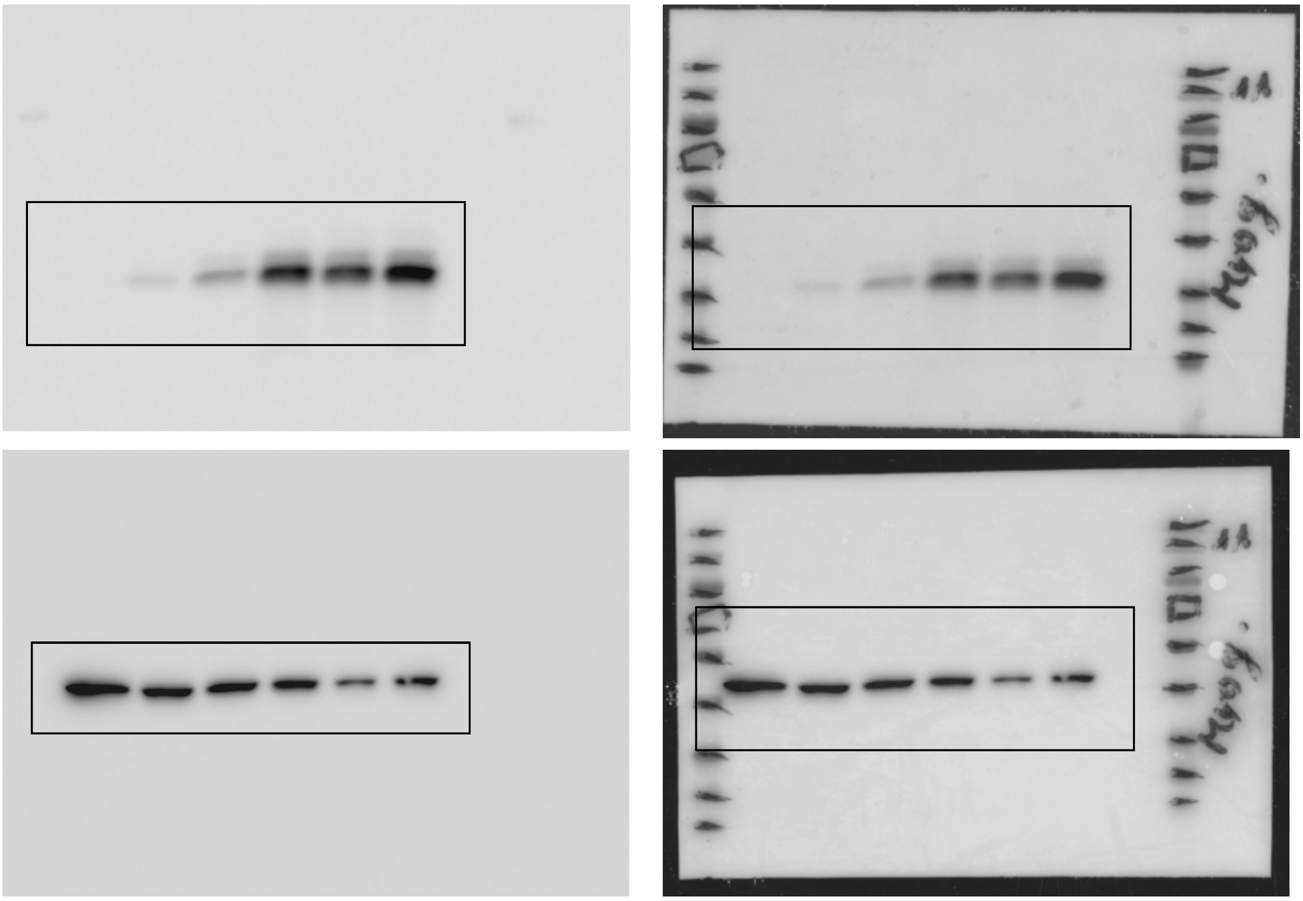

Fig. 1c

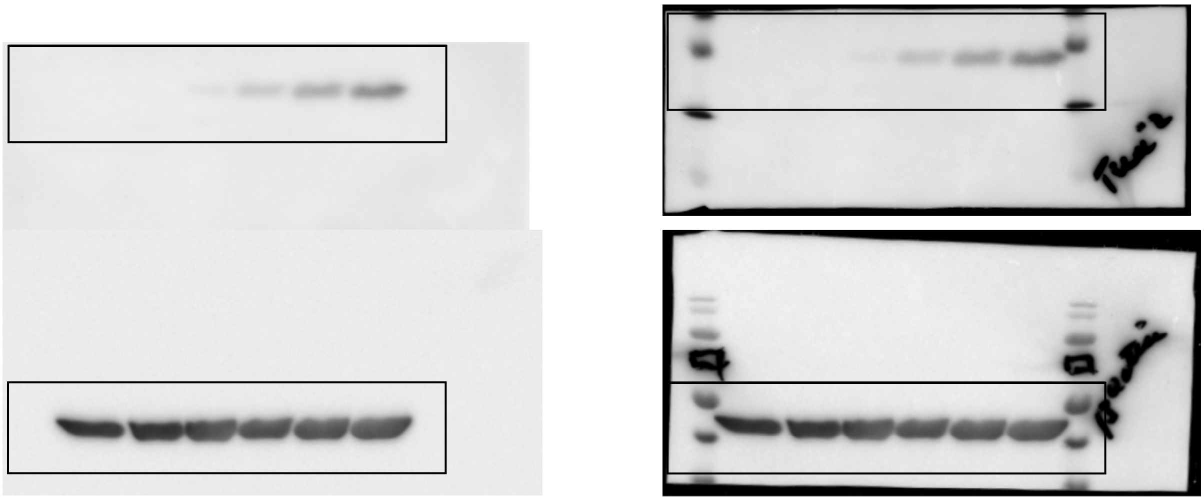

Supplementary Figure S1. Full-length images for the Western Blots shown in Fig. 1. Bioluminescence images (left) and overlay of the bioluminescence and bright field images (right) are presented.

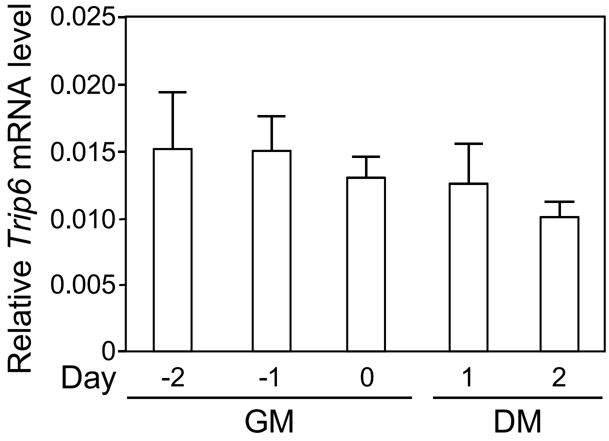

Supplementary Figure S2. *Trip6* mRNA levels do not increase during myoblast differentiation. Total RNA was extracted from C2C12 myoblasts at the indicated day of a differentiation experiment. Day 0 corresponds to the switch from growth medium (GM) to differentiation medium (DM). The relative levels of *Trip6* mRNA were determined by reverse transcription and real-time PCR. Results are plotted relative to the expression of the *Rplp0* gene (mean ± SD of three independent experiments).

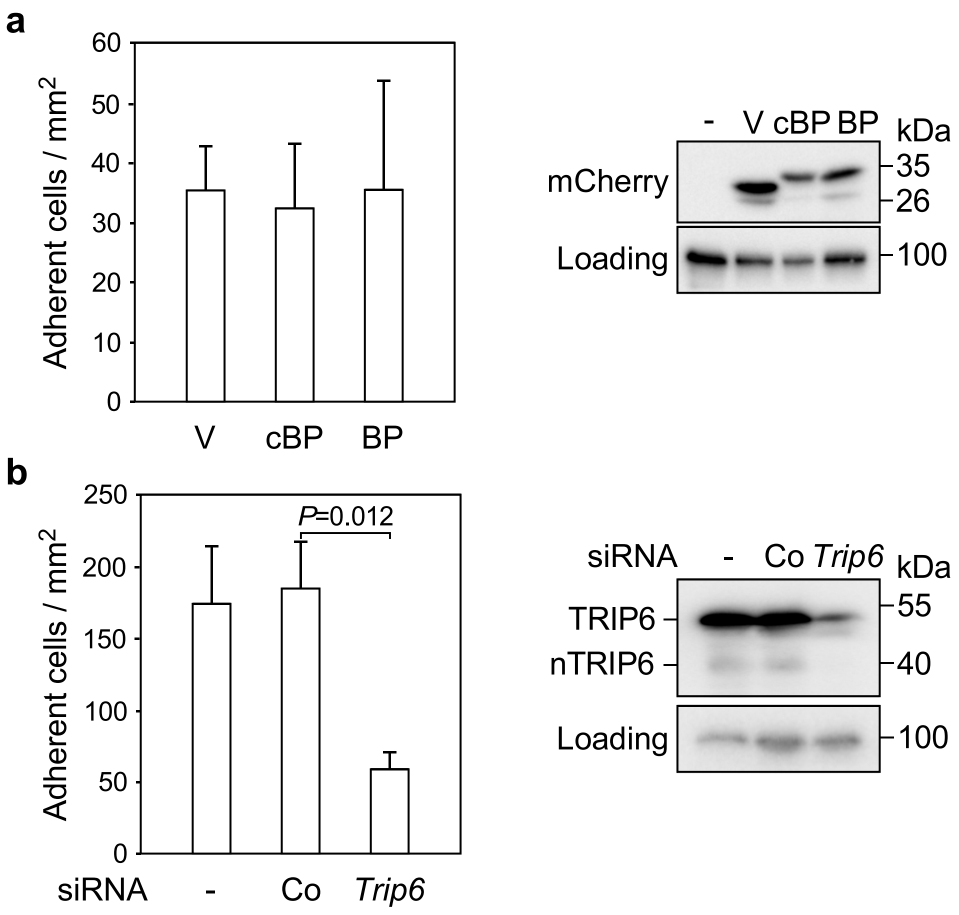

Supplementary Figure S3. The blocking peptide does not interfere with the cytosolic functions of TRIP6. (**a**) C2C12 myoblasts were transfected with either nuclear-targeted mCherry as a vector control (V), the control peptide (cBP) or the blocking peptide (BP) and an adhesion assay was performed 48h later. The number of adhered transfected cells (mCherry positive) is presented as mean ± SD of 3 independent experiments. A representative Western Blot of the cell lysates probed with an anti-mCherry antibody and an anti-GR antibody as a loading control is presented. (**b**) C2C12 myoblasts were either untransfected (-) or transfected with a control siRNA (Co) or an siRNA targeting *Trip6* mRNA. An adhesion assay was performed 48h later and nuclei were stained with DRAQ7. The number of adhered cells is presented as mean ± SD of 3 independent experiments. A representative Western Blot of the cell lysates probed with an anti-TRIP6/nTRIP6 antibody and an anti-GR antibody as a loading control is presented. Full-length blots are presented in Supplementary Figure S4.

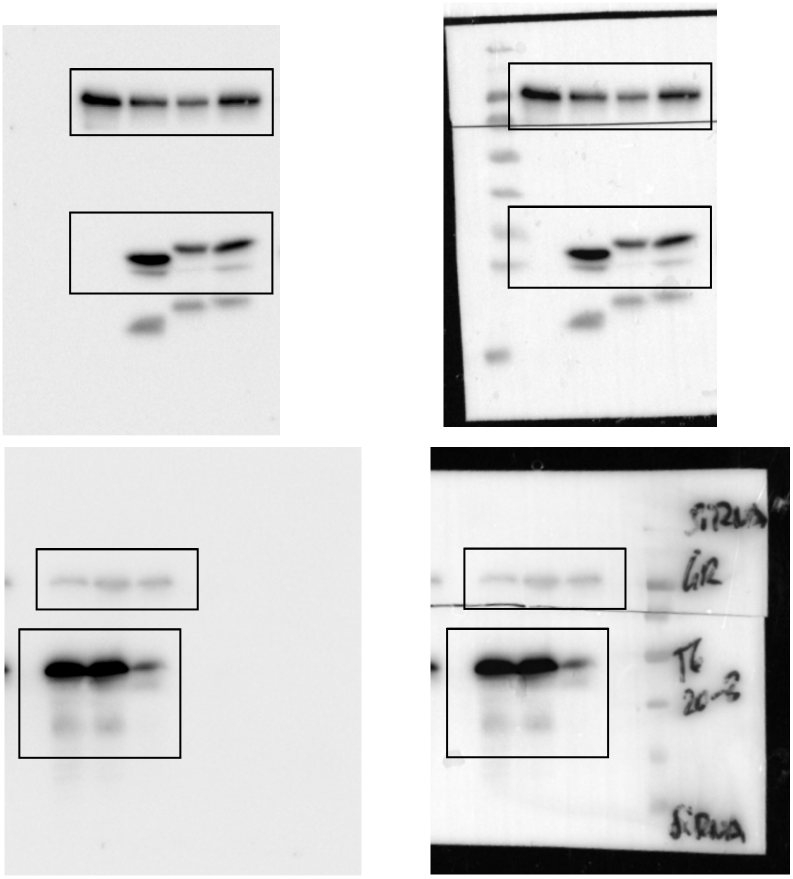

Supplementary Figure S4. Full-length images for the Western Blots shown in Supplementary Fig. S3. Bioluminescence images (left) and overlay of the bioluminescence and bright field images (right) are presented.

Fig. 2d

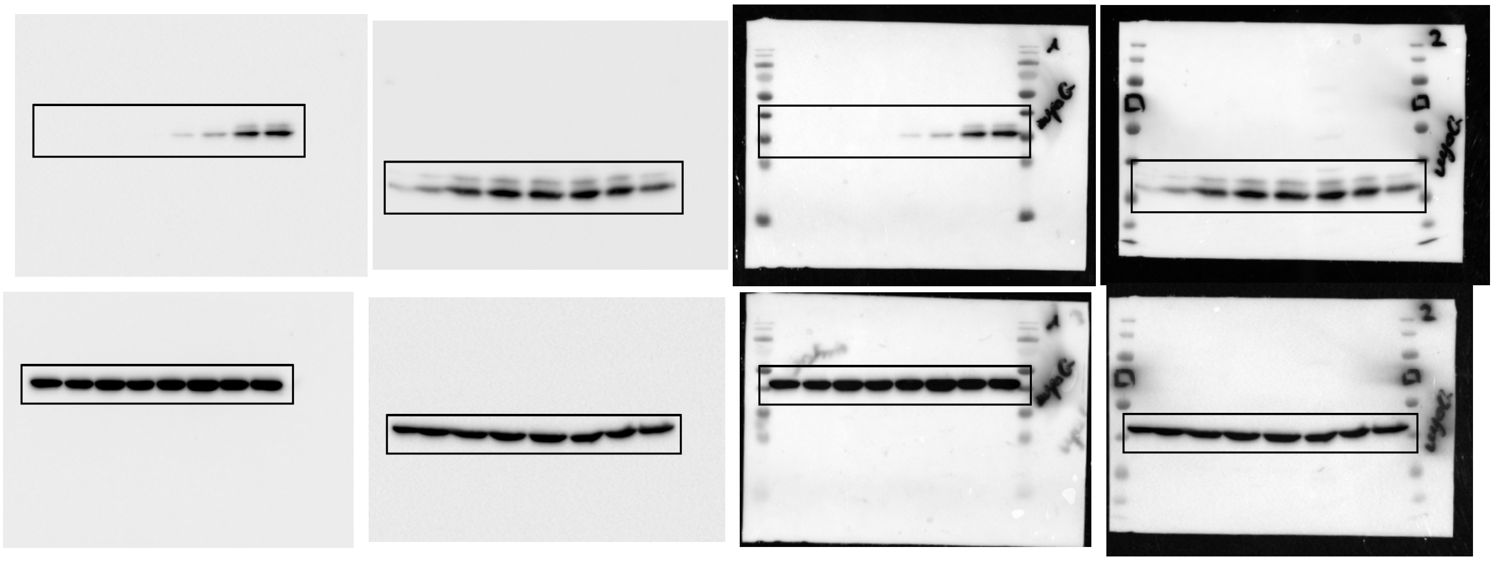

Fig. 2e

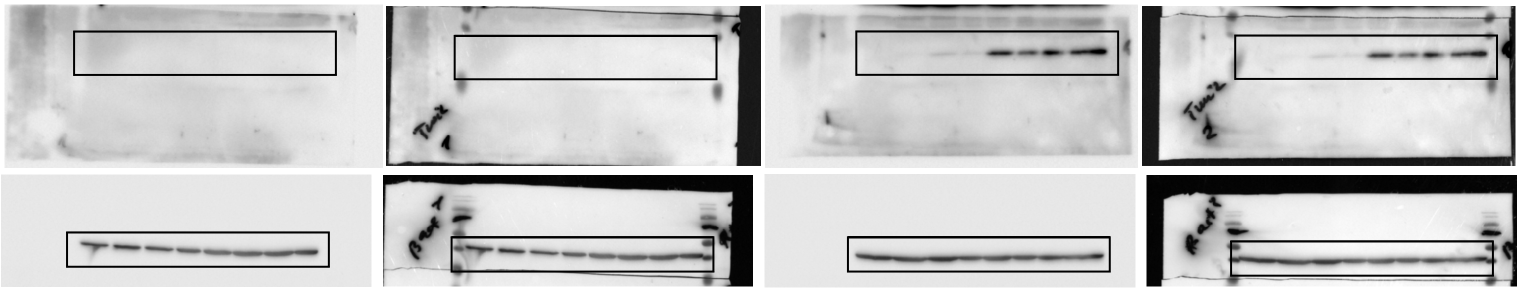

Fig. 2f

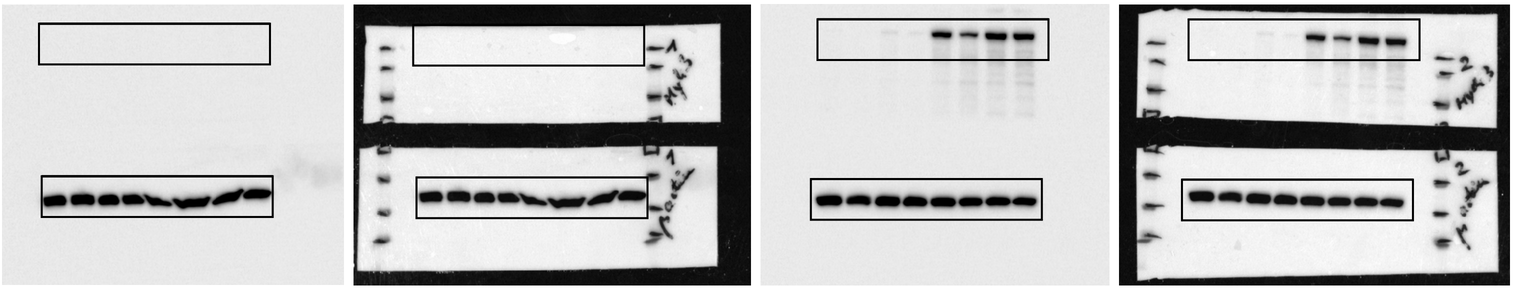

Supplementary Figure S5. Full-length images for the Western Blots shown in Fig. 2. Bioluminescence images (left) and overlay of the bioluminescence and bright field images (right) are presented.

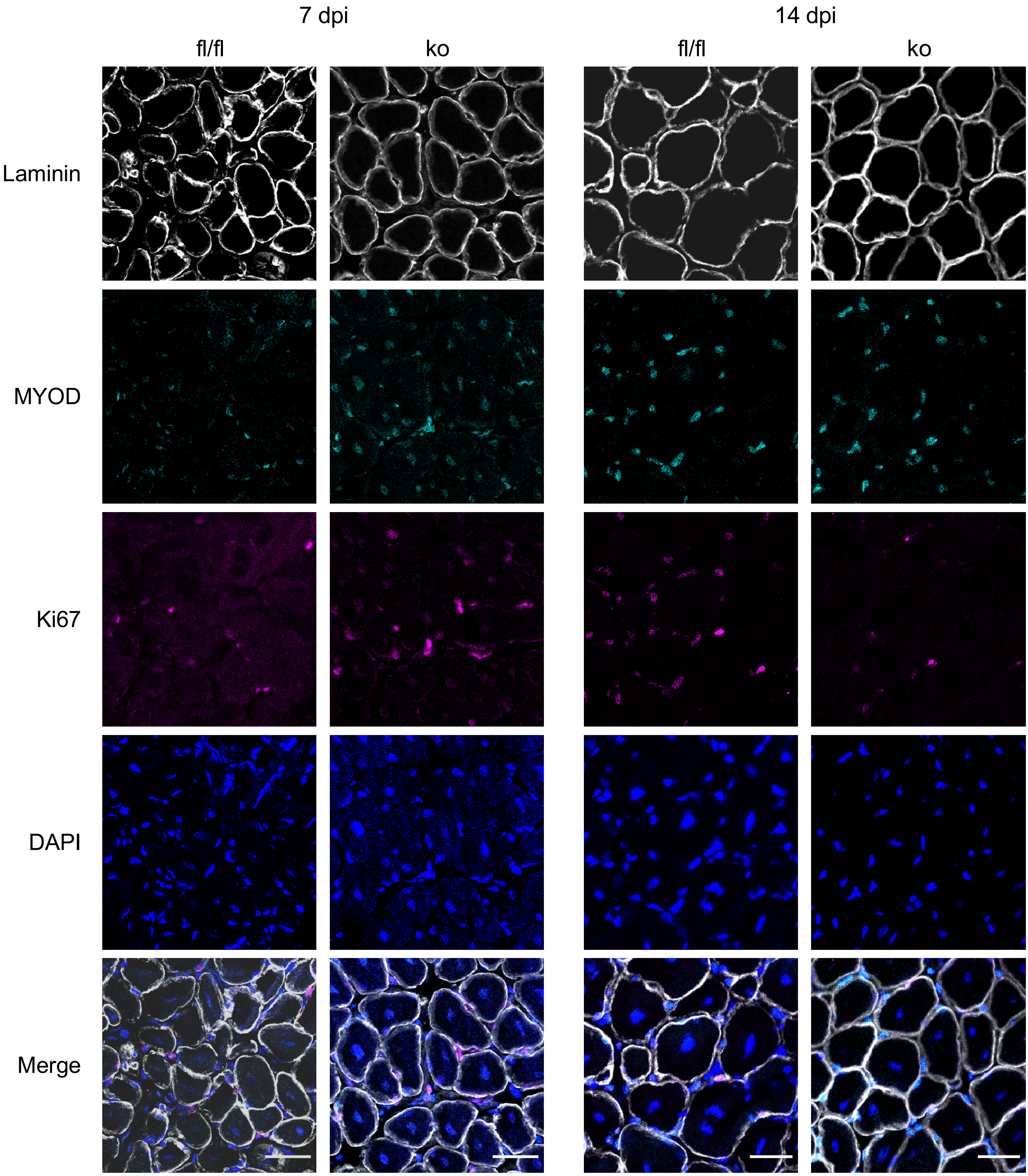

Supplementary Figure S6 - Part 1. Representative images of the experiment presented in Fig. 6. See Fig. 6 legend for details (scale bar: 30 µm).

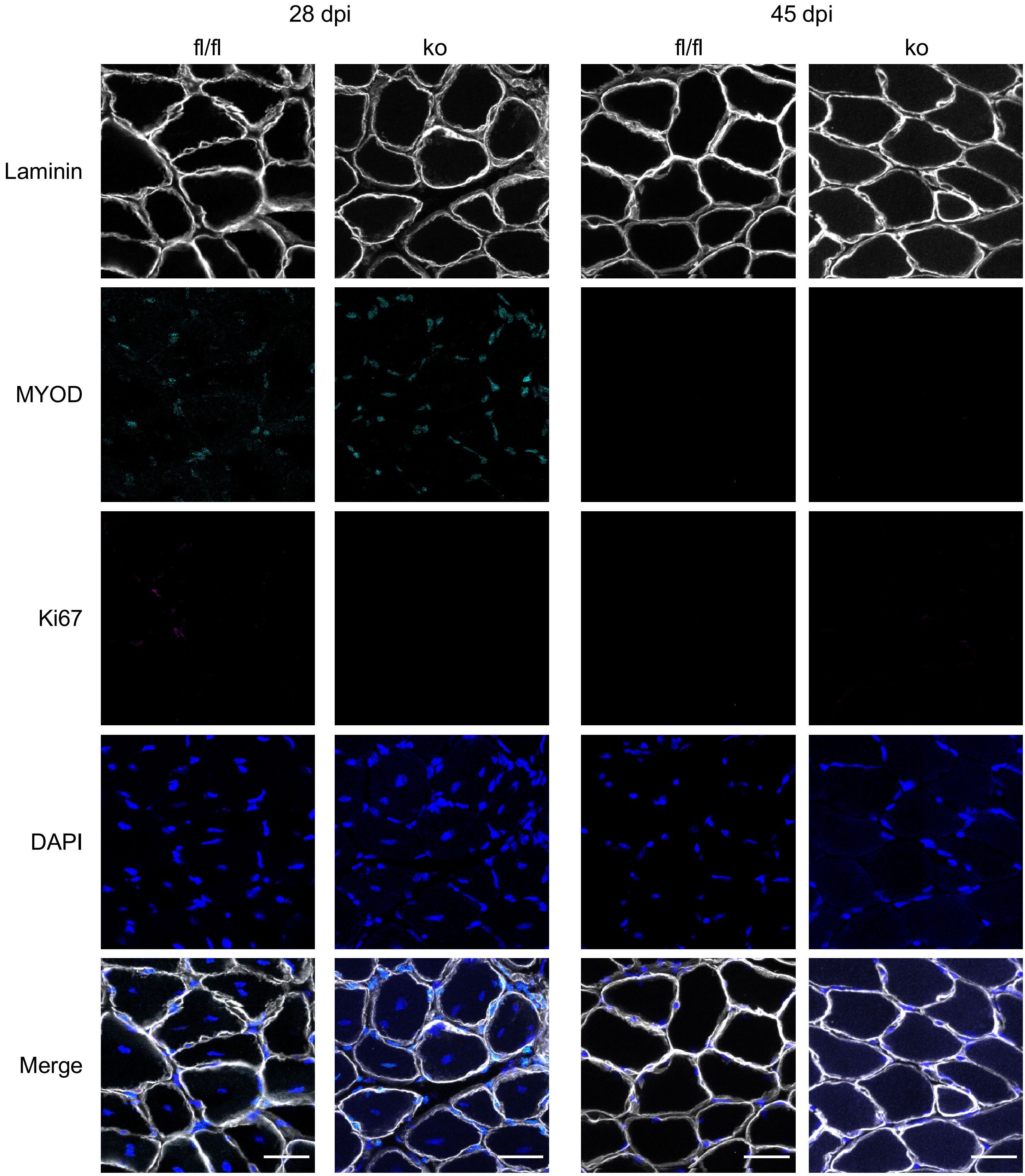

Supplementary Figure S6 - Part 2. Representative images of the experiment presented in Fig. 6. See Fig. 6 legend for details (scale bar: 30 µm).

Supplementary Tables

Supplementary Table S1: siRNA sequences

		Sequence 5' to 3'	Position *
siRNA §	*Trip6*	GUCUGGAUGCUGAGAUAGA	449-467
	dsRed	AGUUCCAGUACGGCUCCAA	

* Positions relative to mouse *Trip6* mRNA sequence (NM_011639.3). § The targeted mRNA sequence is indicated.

Supplementary Table S2: Real-time PCR primers

Gene name	Forward primer (5' to 3')	Reverse primer (5' to 3')
Myog	GAGACATCCCCCTATTTCTACCA	GCTCAGTCCGCTCATAGCC
Rplp0	GGACCCGAGAAGACCTCCTT	GCACATCACTCAGAATTTCAATGG
Tnni2	CATGGAGGTGAAGGTGCAGA	CTCTTGAACTTGCCCCTCAGG

Supplementary Table S3: Genotyping primers

		Sequence 5' to 3'
Pax7 allele	gtPax7CreERT2For	GCTGCTGTTGATTACCTGGC
	gtPax7CreERT2wtRev	CTGCACTGAGACAGGACCG
	gtPax7CreERT2mRev	CAAAAGACGGCAATATGGTG
Trip6 allele	gtTrip6flFor	TCACCTTTTCTCCCTTGCCTGCCTG
	gtTrip6flRev	GGTACCCCCGGAGGCTGATAACAG
